# Bi-specific antibody engagers for cancer immunotherapy

**DOI:** 10.21203/rs.3.rs-4792057/v1

**Published:** 2024-08-01

**Authors:** Hidde Ploegh, Xin Liu, Camille Le Gall, Ryan Alexander, Ella Borgman, Thomas Balligand

**Affiliations:** Boston Children’s Hospital; Boston Children’s Hospital; Radboud Institute for Molecular Life Sciences, Radboud University Medical Center; Boston Children’s Hospital, Harvard Medical School; Boston Children’s Hospital, Harvard Medical School; Boston Children’s Hospital, Harvard Medical School

## Abstract

Bispecific antibody engagers are fusion proteins composed of a nanobody that recognizes immunoglobulin kappa light chains (${\text{VHH}}_{\text{kappa}}$) and a nanobody that recognizes either CTLA-4 or PD-L1. These fusions show strong antitumor activity in mice through recruitment of polyclonal immunoglobulins independently of specificity or isotype. In the MC38 mouse model of colorectal carcinoma, the anti-CTLA-4 ${\text{VHH-VHH}}_{\text{kappa}}$ conjugate eradicates tumors and reduces the number of intratumoral regulatory T cells. The anti-PD-L1 ${\text{VHH-VHH}}_{\text{kappa}}$ conjugate is less effective in the MC38 model, whilst still outperforming an antibody of similar specificity. The potency of the anti-PD-L1 ${\text{VHH-VHH}}_{\text{kappa}}$ conjugate was strongly enhanced by installation of the cytotoxic drug maytansine or a STING agonist. The ability of such fusions to engage the Fc-mediated functions of all immunoglobulin isotypes is an appealing strategy to further improve on the efficacy of immune checkpoint blockade, commonly delivered as a monoclonal immunoglobulin of a single defined isotype.

## Introduction

Antibodies exert their protective and therapeutic effects by different mechanisms.^1^ Ligation of surface receptors by antibodies can either activate or inhibit the targeted structure. By occluding features essential to a protein’s function, antibodies can block receptor-ligand interactions, impede access to substrates or thwart a pathogen’s route of access to the host. The variable regions of the immunoglobulin (Ig) heavy and light chains account for the specificity of recognition. An antibody’s effector functions, carried in its Fragment crystallizable (Fc) region, enable cytotoxicity that is either complement-mediated (complement-dependent cytotoxicity; CDC) or carried out by cells that bear Fc receptors (FcR) of the appropriate specificity (antibody-dependent cell-mediated cytotoxicity; ADCC). Phagocytosis of Ig-decorated structures such as viruses, bacteria or cell remnants is likewise mediated by Fc receptors (antibody-dependent cell-mediated phagocytosis; ADCP).^2,3^

Therapeutic antibodies are typically deployed as a single isotypic variant, such as an IgG1 or IgG4.^4^ Features that allow engagement of Fc receptors may be inactivated by introduction of suitable mutations or left intact, depending on the mechanism of action of the therapeutic agent^.3^ The Fc portions of the different Ig isotypes show distinct activities with respect to complement activation and Fc receptor engagement, resulting in different outcomes: whereas the Fc portion of an IgE molecule is particularly good at inducing mast cell activation,^5^ the Fc portion of IgM is superior at activation of the complement cascade,^6^ while IgG can trigger ADCC.^3^ Humans and the clinically relevant model organisms all possess Igs of the IgM, IgG, IgA and IgE classes (isotypes) as well as receptors for their Fc portions. Presumably, evolution selected for the maintenance of the different Ig isotypes and the Fc receptors specific for them to maximize immune protection.

When considering the therapeutic mechanisms implicated in immunotherapy of cancer, both receptor blockade and Fc-mediated effector functions can play a role.^7,8^ In addition to the properties intrinsic to Igs, further functionalization, for example with cytotoxic drugs to create antibody-drug conjugates, can potentiate their therapeutic efficacy.^9^ The concept of checkpoint blockade has made the use of monoclonal antibodies against cytotoxic T-lymphocyte-associated protein 4 (CTLA-4) and the programmed cell death protein-1 and its ligand (PD-1/PD-L1) a cornerstone of cancer immunotherapy.^10,11^ These forms of immunotherapy rely on monoclonal antibodies, typically of the IgG class. This means that, for a given therapeutic antibody, only a single type of Fc portion determines its therapeutic efficacy. For anti-CTLA-4 immunotherapy, both receptor blockade and Fc receptor (FcR) engagement contribute to its efficacy.^12–17^ The situation is less clear-cut for interference with the PD-1/PD-L1 axis, where direct interference with the PD-1/PD-L1 interactions is believed to dominate over Fc-mediated effects, but the latter has been documented as well.^18^

We have shown that nanobodies, the recombinantly expressed variable regions of heavy chain-only antibodies (VHHs),^19,20^ can be used to target CTLA-4 and PD-L1.^16,21^ Perhaps not surprisingly, administration of nanobodies as immunotherapy is not particularly effective in the absence of an Fc portion. For CTLA-4, the installation of an Fc portion on such nanobodies is required for their antitumor properties.^16^

**Figure 1:**
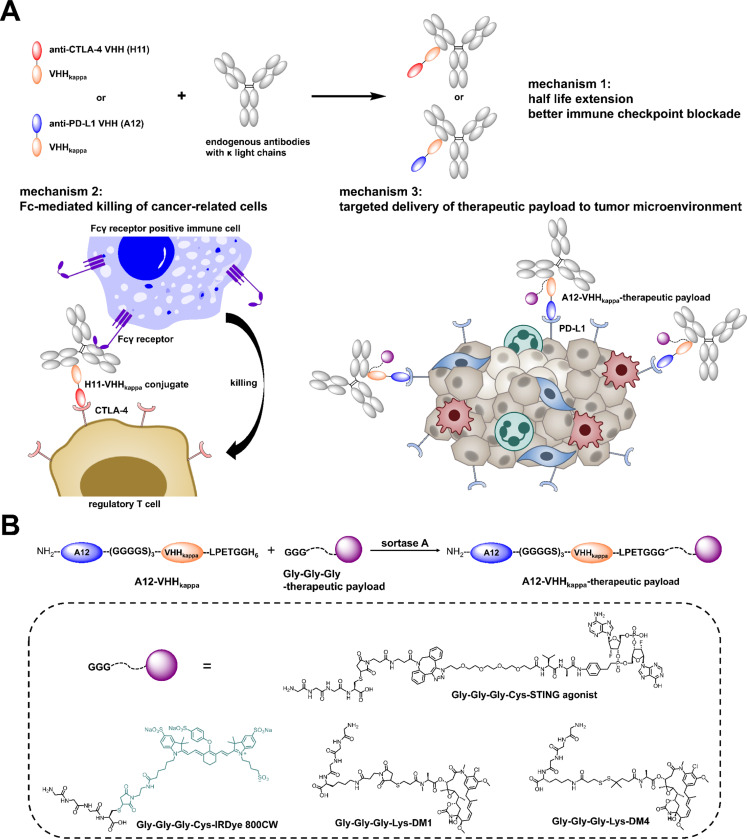
Recruitment of polyclonal immunoglobulins using nanobody-based immune checkpoint inhibitor-${\text{VHH}}_{\text{kappa}}$ conjugates: proposed mechanism of action. (A) Mechanism of action for ${\text{VHH}}_{\text{kappa}}$-conjugated anti-CTLA-4 VHH (H11) and anti-PD-L1 VHH (A12) as anti-cancer immunotherapies: (1) the ${\text{H11-VHH}}_{\text{kappa}}$ or ${\text{A12-VHH}}_{\text{kappa}}$ conjugate binds to kappa light chains of polyclonal immunoglobulins, regardless of specificity or isotype, thereby prolonging the circulatory half-life of the conjugates; (2) the ${\text{H11-VHH}}_{\text{kappa}}$ conjugate recruits immunoglobulins to mediate Fc receptor-dependent depletion of CTLA-4-positive T regulatory cells; (3) the ${\text{A12-VHH}}_{\text{kappa}}$ conjugate can be further modified for delivery of drugs to the tumor microenvironment by targeting PD-L1, overexpressed by tumor and myeloid cells. (B) The triglycine-modified therapeutic payload is site-specifically attached to the C-terminus of ${\text{A12-VHH}}_{\text{kappa}}$ conjugate through a sortase reaction. These payloads include a near-infrared dye imaging agent, cytotoxic drugs, and immunostimulatory drugs.

Here we used a radically different approach to exploit Fc effector functions. We applied a strategy that enables engagement of all Ig isotypes, regardless of their specificity, to exploit the properties associated with the diverse Fc portions of all Igs (Fig. 1A). We showed that nanobodies that recognize Ig light chains (${\text{VHH}}_{\text{kappa}}$) can be functionalized with small molecules to enforce recruitment to virus-infected cells of polyclonal Igs, regardless of isotype or specificity.^22^ We produced a conjugate of ${\text{VHH}}_{\text{kappa}}$ with the influenza virus neuraminidase inhibitor zanamivir to eradicate cells infected with influenza virus. These conjugates afforded protection against a challenge with a lethal dose of virus, even when given several days after infection. We found that zanamivir could be exchanged for a virus-specific nanobody to yield a conjugate that likewise protected against influenza infection. The surprising aspect of this finding is the complete lack of a requirement for antigen-specific interactions of the recruited polyclonal Igs: all of the functional activity that eliminates virus-infected cells must be due to their Fc portions. The engagement of antibodies in this manner is conceptually similar to bi-specific T cell engagers (BiTEs), bi-specific constructs that typically comprise a module that recognizes the CD3 ε subunit of the TCR-CD3 complex, coupled to a tumor-specific recognition module.^23,24^ Upon engagement by a BiTE, T cells, regardless of their specificity, are brought in contact with the target cell to be eliminated. When the BiTE is recruited to the tumor cell, it delivers an activation signal to the T cell to deploy its effector functions, such as cytotoxicity and cytokine release.

Here we extend the concept of nanobody-driven recruitment of polyclonal antibodies to tumor models (Fig. 1A). The circulatory half-life of ${\text{VHH}}_{\text{kappa}}$ conjugates is extended by binding to the much larger Igs, enabling stronger immune checkpoint blockade through sustained presence in the tumor microenvironment (TME). Mindful of the importance of Fc receptor engagement for efficacy of anti-CTLA-4 treatment, we wondered whether the recruitment of polyclonal Igs of all isotypes to CTLA-4 positive cells would improve antitumor activity when compared to a conventional monoclonal antibody. We find that this is indeed the case. We see a pronounced antitumor effect of the anti-CTLA-4 ${\text{VHH-VHH}}_{\text{kappa}}$ conjugate in the MC38 model of colorectal cancer, which we attribute primarily to depletion of regulatory T (Treg) cells in the TME. At the same time this treatment increases the number of T cells in the tumor. We made a similar conjugate of an anti-PD-L1 nanobody with ${\text{VHH}}_{\text{kappa}}$. The anti-PD-L1 ${\text{VHH-VHH}}_{\text{kappa}}$ conjugate was less effective than the anti-CTLA-4 ${\text{VHH-VHH}}_{\text{kappa}}$ conjugate. Both types of conjugates still outperformed conventional monoclonal anti-CTLA-4 or anti-PD-L1 monoclonal antibodies in the MC38 model. To improve on the anti-PD-L1 ${\text{VHH-VHH}}_{\text{kappa}}$ conjugate, we created drug adducts of the anti-PD-L1 ${\text{VHH-VHH}}_{\text{kappa}}$ conjugate that were more effective than their unmodified counterparts (Fig. 1B). Both cytotoxic drugs and STING agonists increased antitumor activity when included in these conjugates. In the B16-F10/GVAX melanoma model, we found that both the anti-CTLA-4 ${\text{VHH-VHH}}_{\text{kappa}}$ and anti-PD-L1 ${\text{VHH-VHH}}_{\text{kappa}}$ conjugates were effective in controlling tumor growth and improving survival. These findings introduce new possibilities for improvement of immunotherapies.

## Results

### Production of bispecific antibody engagers with retention of affinity and specificity

To arrive at the desired bispecific antibody engagers, we produced bivalent nanobody conjugates (~ 30 kDa) in *E. coli* as genetic fusions of the anti-CTLA-4 VHH (H11) or the anti-PD-L1 VHH (A12) sequences with ${\text{VHH}}_{\text{kappa}}$, respectively (Figure S1 and S2). Each conjugate carries a C-terminal LPETG sortase recognition motif, followed by a polyhistidine (6xHis) tag to allow purification on a nickel-nitrilotriacetic acid (Ni-NTA) matrix. Where indicated, the sortase reaction was used for site-specific installation of triglycine-modified small molecule drug payloads at the C-terminus of the ${\text{A12-VHH}}_{\text{kappa}}$ conjugate (Fig. 1B).^25^ The anti-CTLA-4 VHH H11 binds to murine CTLA-4, blocks its ligand-binding site and inhibits its interaction with B7–1, with stronger inhibitory activity than the anti-CTLA-4 monoclonal antibody 9H10.^16^ The anti-PD-L1 VHH A12 binds to murine PD-L1 with subnanomolar affinity and competes with the anti-PD-L1 monoclonal antibody 10F.9G2 for PD-L1 binding.^21^

**Figure 2:**
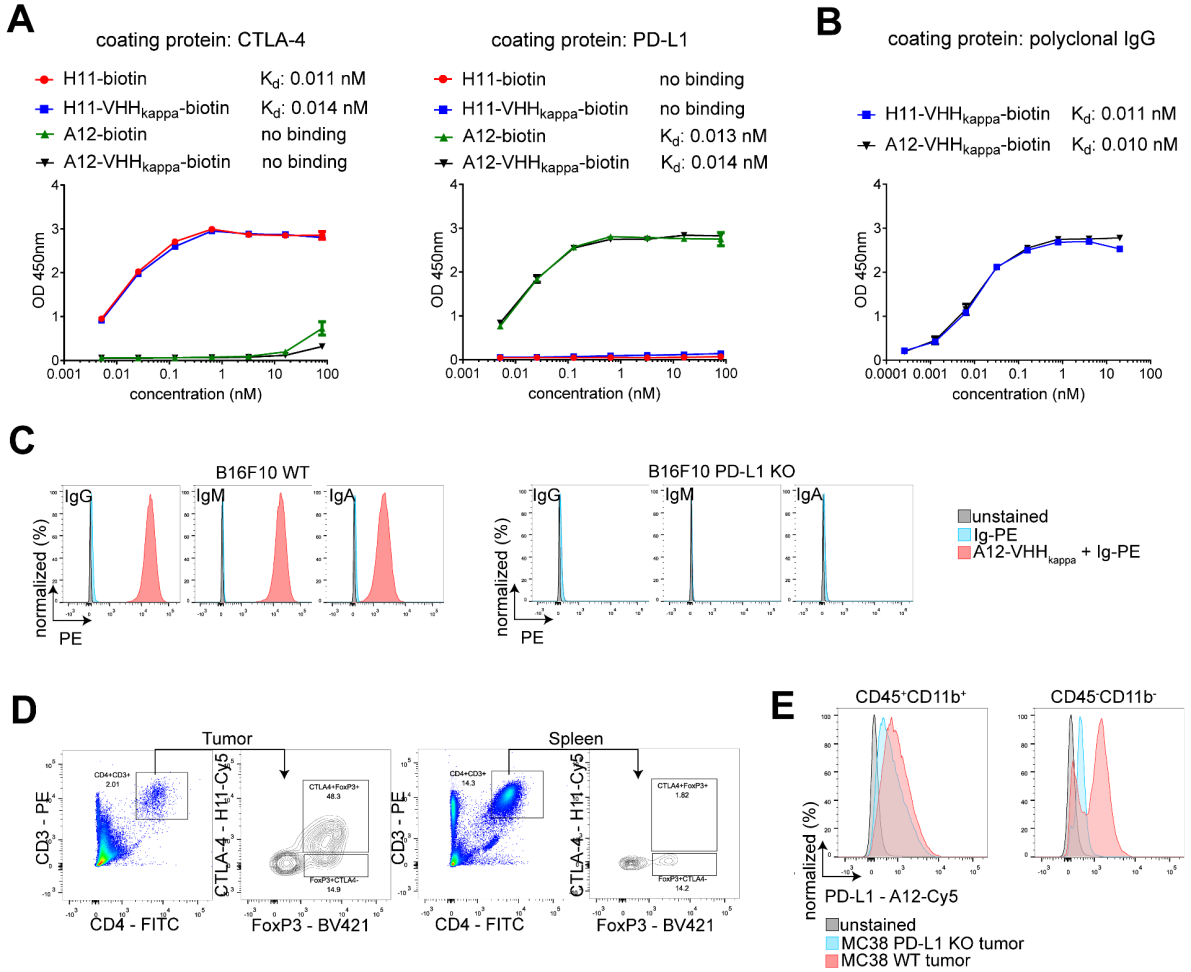
Affinity, specificity and antibody-recruiting capability of the anti-CTLA-4 VHH (H11)-${\text{VHH}}_{\text{kappa}}$ and the anti-PD-L1 VHH (A12)-${\text{VHH}}_{\text{kappa}}$ conjugates. (A) Saturation binding curves of biotinylated H11, A12, ${\text{H11-VHH}}_{\text{kappa}}$ conjugate, and ${\text{A12-VHH}}_{\text{kappa}}$ conjugate to murine CTLA-4 and PD-L1 (Data represent mean ± SD, n = 3). (B) Saturation binding curves of biotinylated ${\text{H11-VHH}}_{\text{kappa}}$ and ${\text{A12-VHH}}_{\text{kappa}}$ conjugates to murine polyclonal IgG (Data represent mean ± SD, n = 3). (C) Flow cytometry confirms that the ${\text{A12-VHH}}_{\text{kappa}}$ conjugate can recruit phycoerythrin (PE)-conjugated mouse IgG, IgM, and IgA, to PD-L1-positive B16-F10 cells. (D) Flow cytometry of MC38 tumor cells, isolated from tumor-bearing C57BL/6 mice and stained with H11-Cy5, reveals CTLA-4 expression on T regulatory cells within the tumor, with only marginal staining observed on splenic T cells. (E) Flow cytometry of cells recovered from PD-L1-positive and PD-L1 knockout MC38 tumors in C57BL/6 mice stained with A12-Cy5 confirms PD-L1 staining on wild-type MC38 cells (CD45^-^) and infiltrating CD11b^+^ immune cells (CD45^+^).

To determine whether H11 and A12 retain their affinity and specificity for their respective targets after conjugation to ${\text{VHH}}_{\text{kappa}}$, we generated saturation binding curves for both the free VHHs and the corresponding ${\text{VHH}}_{\text{kappa}}$ conjugates. Biotinylated ${\text{H11-VHH}}_{\text{kappa}}$ and ${\text{A12-VHH}}_{\text{kappa}}$ showed similar affinity for immobilized CTLA-4 and PD-L1, respectively, as biotinylated H11 and A12 (Fig. 2A). The ${\text{VHH}}_{\text{kappa}}$ portion of these conjugates binds to murine polyclonal IgG without loss of its subnanomolar affinity (Fig. 2B).^22^ The ${\text{A12-VHH}}_{\text{kappa}}$ conjugate recruited phycoerythrin (PE)-conjugated murine antibodies upon binding to PD-L1-positive B16-F10 cells. IgG, IgM, and IgA all bound to B16-F10 cells in the presence of the ${\text{A12-VHH}}_{\text{kappa}}$ conjugate but did not bind to PD-L1 knockout B16-F10 cells (Fig. 2C). We evaluated the ability of the H11 and A12 VHHs to target cancer cells and tumor-infiltrating immune cells isolated from MC38 tumor-bearing C57BL/6 mice. Cyanine5 (Cy5)-labeled H11 binds to CTLA-4 on Tregs extracted from both tumor and spleen, with intratumoral Tregs showing higher CTLA-4 expression than those from the spleen (Fig. 2D). Similarly, A12-Cy5 binds to PD-L1 on wild-type MC38 cells (CD45^−^) and infiltrating CD11b^+^ immune cells (CD45^+^) from both wild-type and PD-L1 knockout MC38 tumors (Fig. 2E). We conclude that the fusion proteins retain the specificity and affinity of their component parts.

### The anti-CTLA-4 VHH (H11)-${\text{VHH}}_{\text{kappa}}$ conjugate exhibits potent antitumor activity by depleting intratumoral regulatory T cells

**Figure 3:**
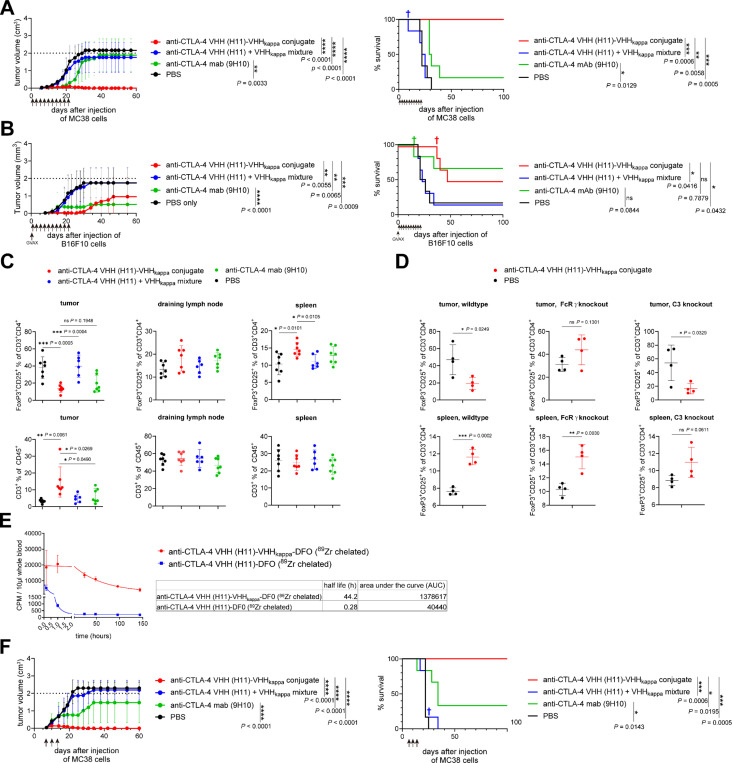
The anti-CTLA-4 VHH (H11)-${\text{VHH}}_{\text{kappa}}$ conjugate shows better *in vivo* antitumor activity than the anti CTLA-4 antibody 9H10 or H11 VHH alone. (A) Tumor growth and survival curves for MC38 tumor-bearing mice (n = 6) treated intraperitoneally with the indicated VHHs at 5 mg/kg on day 1 post-tumor injection, followed by 3 times a week for 3 weeks as indicated by the arrows. † Mouse died from an unknown cause. (B) Tumor growth and survival curves for B16-F10 tumor-bearing mice (n = 6) treated intraperitoneally with the indicated VHHs at 5 mg/kg on day 1 post-tumor injection, followed by 3 times a week for 3 weeks as indicated by the arrows. Mice were vaccinated with GM-CSF–secreting B16 cells (GVAX) on day 0. † Mice were sacrificed due to severe ulceration of their tumors. (C) Changes in T cell populations after treatment with the (H11)-${\text{VHH}}_{\text{kappa}}$ conjugate. MC38 tumor-bearing mice were treated intraperitoneally with the indicated VHHs at 5 mg/kg on day 8, 10, 12, and 14 post-tumor injection. Single cell suspensions from tumors, spleens, and draining lymph nodes were analyzed by flow cytometry on day 15 post-tumor injection. The experiment was performed twice, and the data were combined (Data represent mean ± SD, n= 7, unpaired t test). (D) Changes in regulatory T cell populations after treatment with the ${\text{H11-VHH}}_{\text{kappa}}$ conjugate in wild-type, Fc receptor common gamma chain knockout mice, and complement-deficient mice. MC38 tumor-bearing mice were treated intraperitoneally with the indicated VHHs at 5 mg/kg on day 8, 10, 12, and 14 post-tumor injection. Single cell suspensions from tumors and spleens were analyzed by flow cytometry on day 15 post-tumor injection (Data represent mean ± SD, n= 4, unpaired t test). (E) Half-life measurements of ^89^Zr-labelled ${\text{H11-VHH}}_{\text{kappa}}$ conjugate and ^89^Zr-labelled H11 in mice (Data represent mean ± SD, n = 3). (F) Tumor growth and survival curves for MC38 tumor-bearing mice (n = 6) treated intravenously with the indicated VHHs at 5 mg/kg or anti-CTLA-4 antibody 9H10 at 12.5 mg/kg (equimolar) on day 7, 10, and 13 post-tumor injection as indicated by the arrows. † Mouse was sacrificed due to severe ulceration of its tumor.

We examined the *in vivo* antitumor efficacy of the anti-CTLA-4 VHH (H11)-${\text{VHH}}_{\text{kappa}}$ conjugate in the MC38 mouse colon carcinoma and B16-F10 melanoma models (Fig. 3A and 3B). For the MC38 model, mice received a subcutaneous injection of 1 × 10^6^ cells. For the B16-F10 melanoma model, mice were injected subcutaneously with 5 × 10^5^ B16-F10 melanoma cells and 5 × 10^5^ irradiated B16-GVAX cells (dorsal and ventral, respectively). B16-GVAX cells produce granulocyte-macrophage colony-stimulating factor (GM-CSF) and serve as a vaccine when delivered at the time of tumor challenge.^26^

Treatment consisted of three weekly injections over three weeks with 5 mg/kg of the tested VHHs, as indicated in the figures. The ${\text{H11-VHH}}_{\text{kappa}}$ conjugate slowed tumor growth and improved survival (Figs. 3A and 3B, with individual tumor growth curves in Figures S3A and S3B), while a simple mixture of H11 and ${\text{VHH}}_{\text{kappa}}$ lacked antitumor activity. The ${\text{H11-VHH}}_{\text{kappa}}$ conjugate showed superior efficacy in the MC38 model compared to the anti-CTLA-4 mAb 9H10 (Fig. 3A) and showed similar efficacy in the B16-F10 melanoma model (Fig. 3B). The genetic fusion of SD36 to ${\text{VHH}}_{\text{kappa}}$, which targets influenza virus-infected cells, served as a specificity control and lacked antitumor activity (Figure S4). We installed click handles (an azide and a DBCO moiety) at the C-terminus of the two VHHs by sortase-catalyzed transpeptidation, yielding the genetically impossible C-to-C conjugate of ${\text{H11-VHH}}_{\text{kappa}}$ (Figure S5), which showed similar potent antitumor efficacy (Figure S6). The orientation of the VHHs (C to N, C to C) in such fusions is therefore not important for their antitumor properties.

Fc-dependent depletion of Tregs is a major contributor to the antitumor efficacy of anti-CTLA-4 monoclonal antibodies in mice.^12–17^ We analyzed T cell populations recovered from the tumor after treatment with the ${\text{H11-VHH}}_{\text{kappa}}$ conjugate. We saw selective depletion of Tregs within TME (Fig. 3C, upper panels), without affecting Tregs in the draining lymph nodes or spleen. The percentage of total T cells as a fraction of all CD45^+^ cells increased in the TME of animals that received the ${\text{H11-VHH}}_{\text{kappa}}$ conjugate (Fig. 3C, lower panels). We examined the action of the ${\text{H11-VHH}}_{\text{kappa}}$ conjugate in mice deficient in complement component C3 or lacking the FcR common g chain to better distinguish between CDC and ADCC (Fig. 3D). Depletion of intratumoral Tregs still occurred in C3-deficient mice, but this depletion was compromised in FcR common g chain-deficient mice. Treg depletion by the ${\text{H11-VHH}}_{\text{kappa}}$ conjugate was thus attributable predominantly to FcgR-dependent cytotoxicity.

We next investigated the antitumor activity of the ${\text{H11-VHH}}_{\text{kappa}}$ conjugate on established MC38 tumors. The half-life of the ${\text{H11-VHH}}_{\text{kappa}}$ conjugate was extended upon complex formation with the much larger Igs in the circulation (Fig. 3E). Therefore, treatment was set at 5 mg/kg of the VHHs or 12.5 mg/kg, an equimolar amount, of the anti-CTLA-4 antibody 9H10 administered on days 7, 10, and 13 post-tumor injection. The ${\text{H11-VHH}}_{\text{kappa}}$ conjugate outperformed 9H10 in inhibiting tumor growth and achieved 100% survival in mice bearing established tumors (Fig. 3F, with individual tumor growth curves in Figure S3C). An H11 conjugate with an albumin-specific nanobody has been reported.^27^ This conjugate has a similar size and extended circulatory half-life (53 hours) as the ${\text{H11-VHH}}_{\text{kappa}}$ conjugate but lacks Fc-dependent effector functions. Consequently, although the anti-albumin VHH-conjugated H11 demonstrated antitumor activity in the MC38 model, it required a much higher dose (30 mg/kg given intravenously) and a longer treatment period (3 weeks). Half-life extension is therefore not sufficient for antitumor activity and requires Fc engagement for greater efficacy. The difference in antitumor activity between the ${\text{VHH}}_{\text{kappa}}$-conjugated H11 and the anti-albumin VHH-conjugated H11 indicates that Fc-mediated Treg depletion contributes to optimal antitumor activity.

### The anti-PD-L1 VHH (A12)-${\text{VHH}}_{\text{kappa}}$ conjugate accumulates in PD-L1-positive tumors

**Figure 4:**
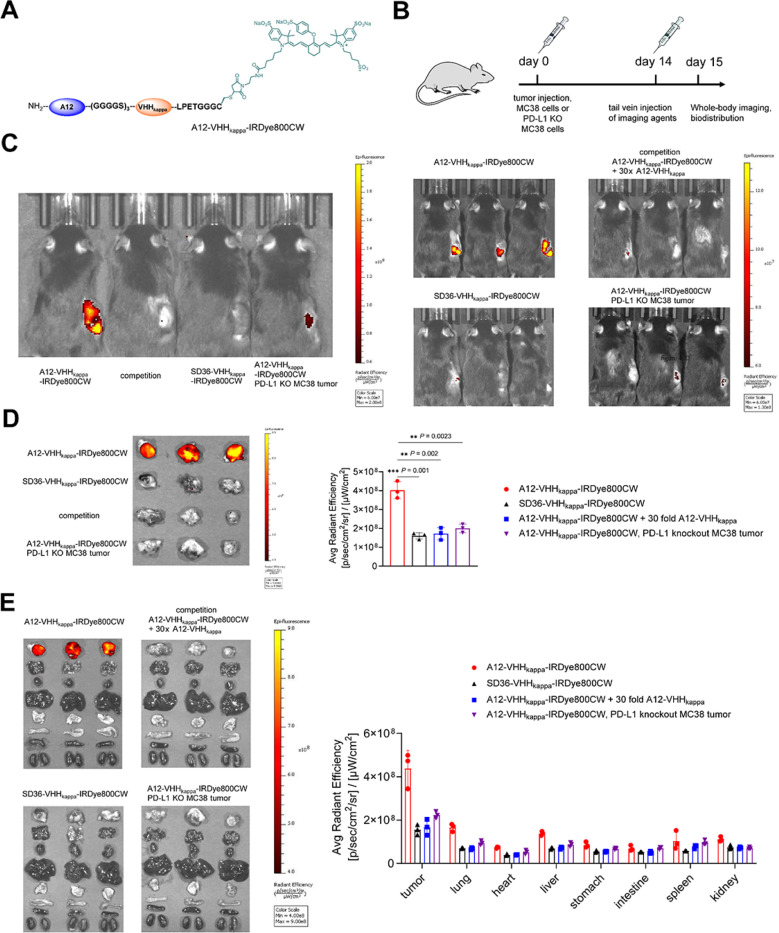
Whole-body imaging and biodistribution of near-infrared dye-labelled anti-PD-L1 VHH (A12)-${\text{VHH}}_{\text{kappa}}$conjugate in MC38 tumor-bearing mice show its PD-L1-dependent accumulation in the tumor. (A) Structure of near-infrared (NIR) dye (IRDye800CW)-labelled ${\text{A12-VHH}}_{\text{kappa}}$ conjugate. (B) Timeline of the imaging experiment. Wild-type and PD-L1 knockout MC38 tumor-bearing mice (n = 3) were treated intravenously with the indicated NIR dye-labelled VHHs (1 nmol) on day 14 post-tumor injection. Imaging was done 24 h later. (C) Whole-body imaging. Left: comparison of various NIR dye-labelled VHHs in tumor-bearing mice; right: individual images of mice from the same treatment group. SD36 is a VHH that recognizes influenza virus hemagglutinin (specificity control). Where indicated (30x), a 30-fold molar excess of unlabeled imaging agent was given as a competitor. (D) Left: *Ex vivo* NIR imaging of tumors from the mice treated with the indicated NIR dye-labelled VHHs; Right: quantification of the NIR dye-labelled VHHs accumulated in the tumors (Data represent mean ± SD, n = 3, unpaired t test). (E) Left: *Ex vivo* NIR imaging of organs from mice treated with the indicated NIR dye-labelled VHHs (from top to bottom: tumor, lung, heart, liver, stomach, intestine, spleen, and kidneys); Right: quantification of accumulation of the NIR dye-labelled VHHs (Data represent mean ± SD, n = 3, unpaired t test).

PD-L1 is overexpressed by tumor cells and myeloid cells in the TME (Fig. 2E).^28^ To investigate whether the ${\text{A12-VHH}}_{\text{kappa}}$ conjugate specifically accumulates in the tumor upon injection, we modified the conjugate with a near-infrared (NIR) dye (IRDye800CW) using a sortase reaction (Fig. 4A, S7, and S8) and performed NIR imaging. Using CRISPR/Cas9, we generated PD-L1 deficient MC38 and B16-F10 cell lines (Figure S9). Mice bearing wild-type and PD-L1 knockout MC38 tumors were injected intravenously with ${\text{A12-VHH}}_{\text{kappa}}$-IRDye800CW on day 14 post-tumor injection. Imaging was performed 24 hours after injection of the imaging agents (Fig. 4B). ${\text{A12-VHH}}_{\text{kappa}}$-IRDye800CW treated mice bearing wild-type MC38 tumors showed greater uptake of the NIR dye in tumors than other groups, including mice bearing PD-L1 deficient tumors (Fig. 4C and 4D). This uptake was blocked by a 30-fold molar excess of the unlabeled ${\text{A12-VHH}}_{\text{kappa}}$ conjugate. The uptake of ${\text{A12-VHH}}_{\text{kappa}}$-IRDye800CW is therefore PD-L1-dependent. The ${\text{SD36-VHH}}_{\text{kappa}}$-IRDye800CW, which targets influenza hemagglutinin and served as a specificity control, showed similar accumulation of dye as the other control groups. The much reduced uptake of ${\text{A12-VHH}}_{\text{kappa}}$-IRDye800CW in the PD-L1 deficient tumors indicates that the MC38 tumor cells, rather than PD-L1-positive myeloid cells, are primarily responsible for the imaging agent’s uptake.Biodistribution analysis showed that ${\text{A12-VHH}}_{\text{kappa}}$-IRDye800CW specifically accumulated in the tumor and not in other major organs (Fig. 4E), further supporting the suitability of the ${\text{A12-VHH}}_{\text{kappa}}$ conjugate for targeted delivery of various drugs to the tumor.

### The antitumor activity of anti-PD-L1 VHH (A12)-${\text{VHH}}_{\text{kappa}}$ requires PD-L1 expression by tumor cells

**Figure 5:**
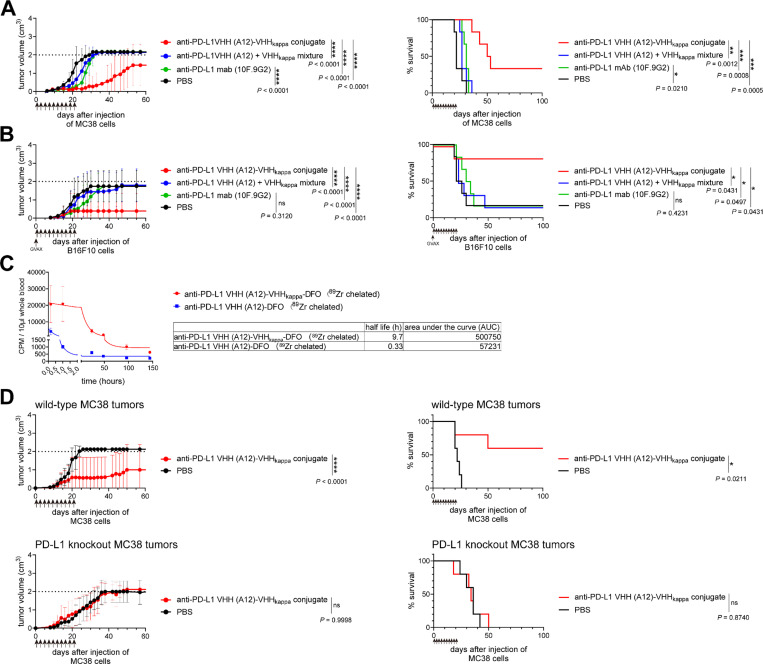
The anti-PD-L1 VHH (A12)-${\text{VHH}}_{\text{kappa}}$ conjugate has superior *in vivo* antitumor activity compared to anti-PD-L1 antibody 10F.9G2 or A12 VHH alone. (A) Tumor growth and survival curves for MC38 tumor-bearing mice (n = 6) treated intraperitoneally with the indicated VHHs at 5 mg/kg on day 1 post-tumor injection, followed by 3 times a week for 3 weeks as indicated by the arrows. (B) Tumor growth and survival curves for B16-F10 tumor-bearing mice (n = 6) treated intraperitoneally with the indicated VHHs at 5 mg/kg on day 1 post-tumor injection, followed by 3 times a week for 3 weeks, as indicated by the arrows. Mice were vaccinated with GM-CSF-secreting B16 cells (GVAX) on day 0. (C) Half-life measurements of ^89^Zr-labelled ${\text{A12-VHH}}_{\text{kappa}}$ conjugate and ^89^Zr-labelled A12 in mice (Data represent mean ± SD, n = 3). (D) Tumor growth and survival curves for wild-type and PD-L1 knockout MC38 tumor-bearing mice (n = 5) treated intraperitoneally with the indicated VHHs at 5 mg/kg on day 1 post-tumor injection, followed by three times a week for 3 weeks as indicated by the arrows.

We examined the *in vivo* efficacy of the anti-PD-L1 VHH (A12)-${\text{VHH}}_{\text{kappa}}$ conjugate using the MC38 mouse colon carcinoma and B16-F10 melanoma models (Fig. 5A and 5B), with the experimental setup described earlier. The ${\text{A12-VHH}}_{\text{kappa}}$ conjugate slowed tumor growth and improved survival (Fig. 5A and 5B, with individual tumor growth curves in Figures S10A and S10B), while a simple mixture of A12 and ${\text{VHH}}_{\text{kappa}}$ lacked antitumor activity. The ${\text{A12-VHH}}_{\text{kappa}}$ showed superior efficacy in both MC38 and B16-F10 models compared to the anti-PD-L1 mAb 10F.9G2 (Fig. 5A and 5B). The C-to-C conjugate of ${\text{A12-VHH}}_{\text{kappa}}$ also showed potent antitumor efficacy. (Figure S11 and S12). Unlike treatment with the anti-CTLA-4 VHH (H11)-${\text{VHH}}_{\text{kappa}}$ conjugate, the ${\text{A12-VHH}}_{\text{kappa}}$ conjugate induced tumor rejection in only a fraction of the animals. We found that the half-life of the ${\text{A12-VHH}}_{\text{kappa}}$ conjugate was extended upon complex formation with circulating Igs (9.7 h, Fig. 5C), but to a lesser extent than that of the ${\text{H11-VHH}}_{\text{kappa}}$ conjugate (44.2 h, Fig. 3E). We implanted MC38 wild-type and MC38 PD-L1 knockout cells in C57BL/6 mice and started treatment at day 1 post-injection. Mice bearing tumors lacking PD-L1 failed to respond to treatment (Fig. 5D and S10C). Thus, PD-L1 expression by tumor cells is required for the efficacy of the ${\text{A12-VHH}}_{\text{kappa}}$ conjugate treatment.

#### Improving the antitumor effect of the anti-PD-L1 VHH (A12)-${\text{VHH}}_{\text{kappa}}$ conjugate through production ofcytotoxic drug conjugates

In view of the limited antitumor activity of the ${\text{A12-VHH}}_{\text{kappa}}$ conjugate we generated a ${\text{A12-VHH}}_{\text{kappa}}$ drug adduct. We first functionalized the ${\text{A12-VHH}}_{\text{kappa}}$ conjugate with maytansine, a cytotoxic drug that binds to tubulin and inhibits microtubule assembly,^29^ for targeted delivery of this toxin to the TME. Two triglycine-modified maytansine derivatives, DM1 (with a non-cleavable linker, Figure S13) and DM4 (with a disulfide cleavable linker, Figure S14), were site-specifically conjugated to the C-terminus of the ${\text{A12-VHH}}_{\text{kappa}}$ conjugate using a sortase reaction (Figure S15 and S16). Although B16-F10 murine melanoma cells were less sensitive to maytansine *in vitro* (EC_50_: 47.7 nM) compared to EL4 murine T cell lymphoma cells reported previously (EC50: 3.9 nM),^30^ the DM1-modified ${\text{A12-VHH}}_{\text{kappa}}$ showed comparable cytotoxicity (EC_50_: 48.1 nM) as free maytansine after a three-day incubation with B16-F10 cells with high PD-L1 expression (Figure S17A). This effect is approximately three times stronger than that seen for ${\text{SD36-VHH}}_{\text{kappa}}$-DM1, used as a specificity control. In contrast, when tested on PD-L1 deficient B16-F10 cells, the cytotoxicity of ${\text{A12-VHH}}_{\text{kappa}}$-DM1 was reduced to a level similar to that of ${\text{SD36-VHH}}_{\text{kappa}}$-DM1 (Figure S17A), confirming that the increased uptake of ${\text{A12-VHH}}_{\text{kappa}}$-DM1 depends on surface-expressed PD-L1. The DM4-modified VHHs were also cytotoxic, similar to the DM1-modified VHHs (Figure S17B). As expected, the absolute potency of the conjugates decreased when the incubation time with the cells was reduced from 3 days to 2 hours, a duration chosen to prevent the extracellular reduction of disulfide bonds in the DM4-modified VHHs.

**Figure 6:**
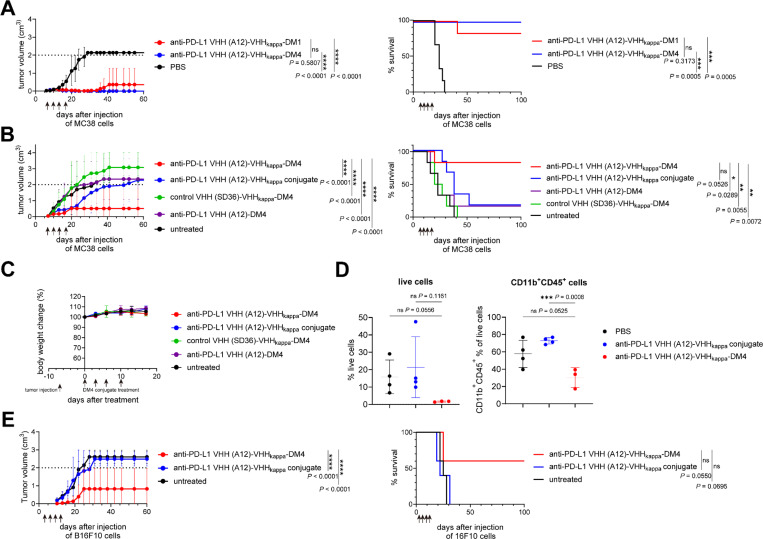
Targeted delivery of the cytotoxicity drug maytansine enhances antitumor activity of the anti-PD-L1 VHH (A12)-${\text{VHH}}_{\text{kappa}}$ conjugate. (A) Tumor growth and survival curves for MC38 tumor-bearing mice (n = 6) treated intravenously with ${\text{A12-VHH}}_{\text{kappa}}$-DM1 or ${\text{A12-VHH}}_{\text{kappa}}$-DM4 at 5 mg/kg on day 7, 10,13, and 17 post-tumor injection as indicated by the arrows. (B) Tumor growth and survival curves for MC38 tumor-bearing mice (n = 6) treated intravenously with indicated VHH-DM4 conjugates at 5 mg/kg on day 7, 10,13, and 17 post-tumor injection as indicated by the arrows. (C) Body weight curves for the mice used in panel B (Data represent mean ± SD, n = 6). (D) Percentage of live cells and CD11b^+^ lymphocytes harvested from the tumors in mice treated with ${\text{A12-VHH}}_{\text{kappa}}$-DM4 or ${\text{A12-VHH}}_{\text{kappa}}$ conjugate. MC38 tumor-bearing mice were treated intravenously with the indicated VHH conjugates at 5 mg/kg on day 11 and 13 post-tumor injection (Data represent mean ± SD, n = 3 or 4, unpaired t test). (E) Tumor growth and survival curves for B16-F10 tumor-bearing mice (n = 5) treated intravenously with indicated VHH-DM4 conjugates at 5 mg/kg on day 3, 6, 9, and 13 post-tumor injection as indicated by the arrows.

Both DM1- and DM4-modified ${\text{A12-VHH}}_{\text{kappa}}$ conjugates have antitumor activity in the MC38 models, with the DM4-modified conjugate achieving 100% survival. (Fig. 6A and S18A). We used the DM4-modified VHHs for further experiments. Treatment with the ${\text{A12-VHH}}_{\text{kappa}}$ conjugate seven days after MC38 cell implantation delayed tumor growth but did not significantly improve the survival rate (Fig. 6B and S18B). In contrast, 5 out of 6 mice treated with ${\text{A12-VHH}}_{\text{kappa}}$-DM4 survived and showed negligible tumor growth. Neither the specificity control VHH (SD36)-${\text{VHH}}_{\text{kappa}}$-DM4 nor A12-DM4 prevented tumor growth. No weight loss was observed in any of the mice that received the conjugates, suggesting a lack of overt toxicity of the DM4 adducts at the given doses (Fig. 6C). Most of the cells harvested from the TME after two doses of ${\text{A12-VHH}}_{\text{kappa}}$-DM4 were non-viable (Fig. 6D). The percentage of CD11b^+^ myeloid cells among the infiltrating immune cells was reduced (Fig. 6D). We tested the antitumor activity of ${\text{A12-VHH}}_{\text{kappa}}$-DM4 on the more aggressive B16-F10 melanoma model in the absence of the GVAX vaccine (Figs. 6E and S18C). We found that modification of the ${\text{A12-VHH}}_{\text{kappa}}$ conjugate with DM4 enhanced its potency against tumor growth and improved survival.

### Targeted delivery of a STING agonist enhances antitumor activity of the anti-PD-L1 VHH (A12)-${\text{VHH}}_{\text{kappa}}$ conjugate

**Figure 7:**
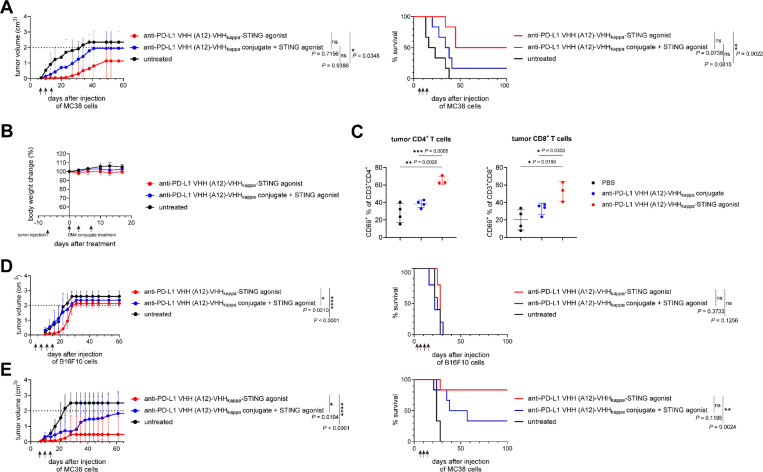
Targeted delivery of a STING agonist enhances antitumor activity of the anti-PD-L1 VHH (A12)-${\text{VHH}}_{\text{kappa}}$conjugate. (A) Tumor growth and survival curves for MC38 tumor-bearing mice (n = 6) treated intravenously with ${\text{A12-VHH}}_{\text{kappa}}$-STING agonist or ${\text{A12-VHH}}_{\text{kappa}}$-conjugate plus free STING agonist (equimolar amount) at 5 mg/kg on day 7, 10, and 14 post-tumor injection as indicated by the arrows. (B) Body weight curves for the mice used in panel A (Data represent mean ± SD, n = 6). (C) Percentage of activated (CD69-positive) CD4^+^ and CD8^+^ T cells harvested from the tumors in mice treated with ${\text{A12-VHH}}_{\text{kappa}}$-STING agonist or ${\text{A12-VHH}}_{\text{kappa}}$ conjugates. MC38 tumor-bearing mice were treated intravenously with the indicated VHH conjugates at 5 mg/kg on day 11 and 13 post-tumor injection. Single cell suspensions from tumors were analyzed by flow cytometry on day 15 post-tumor injection (Data represent mean ± SD, n = 3 or 4, unpaired t test). (D) Tumor growth and survival curves for B16-F10 tumor-bearing mice (n = 5) treated intravenously with ${\text{A12-VHH}}_{\text{kappa}}$-STING agonist or ${\text{A12-VHH}}_{\text{kappa}}$-conjugate plus free STING agonist (equimolar amount) on day 3, 7,11, and 15 post-tumor injection as indicated by the arrows. (E) Tumor growth and survival curves for MC38 tumor-bearing mice (n = 6) treated intravenously with ${\text{A12-VHH}}_{\text{kappa}}$-STING agonist or ${\text{A12-VHH}}_{\text{kappa}}$-conjugate plus free STING agonist (equimolar amount) at 10 mg/kg on day 7, 10, and 14 post-tumor injection as indicated by the arrows.

To facilitate the establishment of an inflammatory environment conducive to an antitumor immune response,^31^ we next created adducts that include a STING agonist.^32^ Targeted delivery of a cyclic dinucleotide STING agonist by means of further modification of the ${\text{A12-VHH}}_{\text{kappa}}$ conjugate (Figure S19) to the TME activated the STING pathway. Treatment with ${\text{A12-VHH}}_{\text{kappa}}$-STING agonist significantly suppressed the growth of MC38 tumors and cured 50% of the mice, demonstrating greater potency than the simple combination of the ${\text{A12-VHH}}_{\text{kappa}}$ conjugate and an equimolar amount of free STING agonist (Fig. 7A and S20A). The lack of significant body weight loss induced by the ${\text{A12-VHH}}_{\text{kappa}}$-STING agonist suggests that this conjugate does not cause systemic inflammation (Fig. 7B). The percentage of activated (CD69-positive) CD4^+^ and CD8^+^ T cells harvested from the tumors of mice treated with two doses of ${\text{A12-VHH}}_{\text{kappa}}$-STING agonist increased significantly (Fig. 7C), indicating that the STING agonist enhances the generation of a T cell response, including the induction of CD8^+^ cytotoxic T cells. The ${\text{A12-VHH}}_{\text{kappa}}$-STING agonist also reduced the growth of the more aggressive B16-F10 tumor more effectively than the ${\text{A12-VHH}}_{\text{kappa}}$ conjugate, but it did not improve the overall survival (Fig. 7D and S20B). Increasing the dose of ${\text{A12-VHH}}_{\text{kappa}}$-STING agonist from 5 mg/kg to 10 mg/kg showed stronger inhibition of tumor growth and improved survival, but with a slight increase in body weight loss (Fig. 7E and S20C).

## Discussion

Bi-specific T cell engagers (BiTes) are in clinical use as antitumor agents. Dual specificity is achieved through the creation of fusions of a T cell recognition module, typically anti-CD3ε, and an antitumor component. The success of BiTes derives from their ability to direct T cells, regardless of their specificity, to the tumor cell and exploit their effector functions.^23,24^ Here we extend the concept of bi-specific engagers by exploiting the ability of fusion proteins to target cells relevant to the antitumor response, and harness the effector functions of all Ig classes in the absence of deliberate immunization. Nanobodies and their derivatives have advantages over the use of conventional antibodies, including: (1) high-yield expression including in prokaryotic expression systems due to their smaller size, superior stability and solubility, as well a lesser dependency on disulfide bond formation and glycosylation; (2) the ability to access epitopes less accessible to conventional antibodies, facilitated by their extended complementarity-determining region 3 (CDR3) loop; (3) low immunogenicity owing to their sequence homology to human immunoglobulin (Ig) V regions and humanization strategies; and (4) their small size and ease of modification, allowing for configurations that are more challenging to achieve with conventional antibodies^.20,33,34^ However, when used as stand-alone therapeutic agents, nanobodies suffer from a short circulatory half-life (< 30 minutes in mice and 0.5–2 hours in humans).^35,36^ Nanobodies obviously lack Fc-dependent effector functions. To overcome the latter shortcomings, a widely used strategy is to conjugate the nanobody of interest with an albumin-specific nanobody^.37,38^ These conjugates extend their half-life by binding to serum albumin in the bloodstream but cannot exert Fc-dependent effector functions. Alternatively, conjugating the nanobody to the Fc fragment can address the lack of Fc-dependent functions, though it compromises some of the nanobody’s desirable properties.^39^ The approach described here achieves both circulatory half-life extension and provides Fc-dependent effector functions, not limited to a single Ig isotype.

Adult mice have the peculiar trait, in that > 95% of all Ig isotypes carry the kappa light chain,^40^ possibly driven by the large number of V k genes that provide the substrate for selection of B cells of the requisite specificity and a l light chain locus of much reduced complexity. In humans, the percentage of circulating Igs carrying kappa light chains is ~ 60%.^41^ We do not anticipate this difference to impact the effectiveness of mouse *vs.* human ${\text{VHH}}_{\text{kappa}}$ conjugates significantly. Given the quantities of circulating Igs in humans and mice, as well as the doses of ${\text{VHH}}_{\text{kappa}}$ conjugates administered, only a small fraction of the circulating Igs will bind the conjugates. The availability of a nanobody specific for human Ig kappa light chains with picomolar binding affinity suggests the potential for using the ${\text{VHH}}_{\text{kappa}}$ conjugate strategy in humans.^42^

We show improved activity of the ${\text{H11-VHH}}_{\text{kappa}}$ conjugate over 9H10 monoclonal antibody in two tumor models. We attribute this improvement to the engagement of all Fc-associated functions, rather than the reliance on a single Fc portion. MC38 is notoriously unresponsive to CTLA-4 blockade,^43,44^ yet all the ${\text{H11-VHH}}_{\text{kappa}}$ conjugate-treated mice successfully rejected tumors. Using H11-Cy5, we confirmed higher levels of CTLA-4 expression on intratumoral Tregs than on splenic Tregs (Fig. 2D) as a possible mechanism for the tumor-specific depletion of Tregs.^45^ Using FcgR-deficient and complement-deficient mice, we demonstrate that Treg depletion is mediated by FcgR. The fraction of Tregs in the spleen increased upon treatment with the ${\text{H11-VHH}}_{\text{kappa}}$ conjugate (Fig. 3C). This was still the case in FcgR - deficient and complement-deficient mice (Fig. 3D), suggesting an Fc-independent mechanism resulting from CTLA-4 signal blockade itself. Our data suggest that polyclonal Ig recruitment and harnessing the diversity of Fc-mediated effects could improve activity of CTLA-4 blockade in human. In MC38 and B16-F10 models, the anti-PD-L1 ${\text{A12-VHH}}_{\text{kappa}}$ conjugate outperformed the reference 10F.9G2 monoclonal antibody but failed to confer complete protection. Flow cytometry of established tumors showed that PD-L1 is expressed on both MC38 tumor cells and tumor-infiltrating CD11b^+^ myeloid cells (Fig. 2E).Because PD-L1 knockout MC38 tumor-bearing mice failed to respond to the ${\text{A12-VHH}}_{\text{kappa}}$ conjugate therapy, expression of PD-L1 on tumor cells is required for therapeutic efficacy, supported by the imaging data (Fig. 4).

We hypothesize that depletion of tumor-associated macrophages in the MC38 model is key to initiate tumor rejection. To improve on the suboptimal activity of the ${\text{A12-VHH}}_{\text{kappa}}$ conjugate, we incorporated a cytotoxic drug, DM4. Treatment with ${\text{A12-VHH}}_{\text{kappa}}$-DM4 improved tumor rejection in both the MC38 and B16-F10 models. MC38 tumors treated with ${\text{A12-VHH}}_{\text{kappa}}$-DM4 contained few live cells and had fewer CD11b^+^CD45^+^ myeloid cells (Fig. 6D), suggesting that A12-VHH kappa-DM4 increased the depletion of tumor-associated macrophages. Delivery of pro-inflammatory signals to the TME promotes tumor clearance.^46,47^ Site-specific incorporation of a STING agonist in the ${\text{A12-VHH}}_{\text{kappa}}$ conjugate improved rejection of MC38 tumors. ${\text{A12-VHH}}_{\text{kappa}}$ STING agonist induced activation of CD8^+^ and CD4 T^+^ cells (Fig. 7C). Incorporation of the STING agonist in the ${\text{A12-VHH}}_{\text{kappa}}$ conjugate delayed tumor growth in the B16-F10 model but failed to improve overall survival.

The greater antitumor activity of the ${\text{VHH}}_{\text{kappa}}$ conjugates when compared with the widely used anti-CTLA-4 monoclonal antibody suggests that different FcR^+^ cells may be responsible for this effect. The contribution of different FcR^+^ cells in response to deployment of the ${\text{VHH}}_{\text{kappa}}$ conjugates requires further exploration. Given the conservation of multiple Ig isotypes across vertebrate species, there may well be an advantage to the engagement of multiple classes of Igs over a single one. We compared the efficacy of monoclonal antibodies specific for CTLA-4 or PD-L1 with the corresponding ${\text{VHH}}_{\text{kappa}}$ conjugates and found the latter to be superior. Perhaps other monoclonal antibodies would perform better than those used here, which have been widely used preclinically, but we consider our results as supporting the value of multiple Ig engagement.

The ${\text{VHH}}_{\text{kappa}}$-nanobody approach can be extended to target additional cancer-specific markers. Eradication of CD20-, HER2-, or EGFR-positive cancer cells by therapeutic antibodies is driven by ADCC/CDC as well as ADCP.^7^ Immunomodulatory reagents can deplete specific immunosuppressive cells to enhance antitumor activities of the remaining T cells. Appealing targets for immunomodulation by ${\text{VHH}}_{\text{kappa}}$ conjugates include OX40, CD25, GITR, CD47 and CD73.^7,8,48^ Our findings support the pursuit of bispecific antibody engagers to target additional cancer-specific and immunomodulatory markers.
